# Leukocytoclastic Vasculitis With Eosinophilia in a Patient Receiving Dupilumab Therapy

**DOI:** 10.7759/cureus.84252

**Published:** 2025-05-16

**Authors:** Alex M Wandler, Jonathan M Joseph, Christopher Haas

**Affiliations:** 1 Department of Dermatology, Louisiana State University Health Sciences Center New Orleans, New Orleans, USA

**Keywords:** atopic disease, drug-induced eosinophilia, dupilumab dermatologic reaction, eosinophilic dermatosis, leukocytoclastic vasculitis (lcv), prurigo nodularis (pn)

## Abstract

We present the case of a patient with a history of treated hepatitis C virus who was diagnosed with prurigo nodularis and subsequently developed leukocytoclastic vasculitis (LCV) with eosinophilia following the initiation of dupilumab therapy.

A 69-year-old African American female with previously treated hepatitis C virus presented with a papular, pruritic skin eruption initially diagnosed as prurigo nodularis. Despite treatment with oral and topical corticosteroids, she experienced no improvement. Physical examination revealed numerous hyperpigmented papules on the extensor surfaces of both upper extremities, without blisters or bullae.

After starting on dupilumab, she noted minimal improvement and developed new erythematous papules on the right upper extremity. A punch biopsy performed due to ongoing symptoms showed perivascular and interstitial neutrophils with numerous eosinophils, consistent with LCV with eosinophilia. Dupilumab was discontinued, and topical tacrolimus was initiated. One month later, repeat biopsy was consistent with lichen simplex chronicus, suggesting the persistence of a chronic inflammatory state following the resolution of the initial vasculitis.

This patient's findings underscore the importance of recognizing a potential association between dupilumab and the development of eosinophilic conditions in the context of dupilumab therapy. The temporal relationship between the initiation of dupilumab treatment and the onset of eosinophilic LCV suggests a potential association that should be further investigated and should encourage dermatologists to remain cognizant of the development of LCV in the setting of dupilumab therapy.

## Introduction

Dupilumab is a monoclonal immunoglobulin (IgG4) antibody that blocks interleukin-4 (IL-4) and interleukin-13 (IL-13) signaling via IL-4 receptor alpha. It is indicated across specialties for moderate-to-severe atopic dermatitis and other type 2 inflammatory conditions. The most reported adverse effects associated with dupilumab therapy in the context of atopic dermatitis include injection site reactions and conjunctivitis [1]. Less frequently, cutaneous effects have been reported, such as erythema nodosum, eosinophilic granulomatosis with polyangiitis, cutaneous T-cell lymphoma, and leukocytoclastic vasculitis (LCV) [2,3]. Although the relationship between dupilumab and eosinophilic conditions remains unclear, inhibition of IL-4 and IL-13 reduces eosinophil migration and activation, increasing their accumulation in the bloodstream and thus facilitating the development of eosinophilic conditions [4].

Pertinent to this patient’s presentation, LCV is a form of small-vessel vasculitis that predominantly affects the skin and is characterized histologically by neutrophilic infiltration and nuclear debris within vessel walls. The etiology of LCV is diverse and may involve immune complex deposition, antibody-mediated processes, underlying systemic diseases, infections, malignancy, or drug exposures. Traditionally, drug-induced LCV has been associated with antibiotics and non-steroidal anti-inflammatory drugs (NSAIDs); however, more recent reports suggest that biological therapies - including tumor-necrosis factor (TNF) inhibitors, monoclonal antibodies, and immune checkpoint inhibitors - may also act as potential triggers [5].

Clinically, LCV most commonly presents with palpable purpura, often accompanied by pain, pruritus, or a burning sensation. Although systemic manifestations - such as fever, arthritis, and gastrointestinal or renal involvement - may occur, cutaneous findings remain the most common, especially in cases associated with TNF inhibitors and other biological therapies [5].

In this case, we present a patient with prurigo nodularis who developed LCV with eosinophilia following the initiation of dupilumab therapy.

## Case presentation

**Figure 1:**
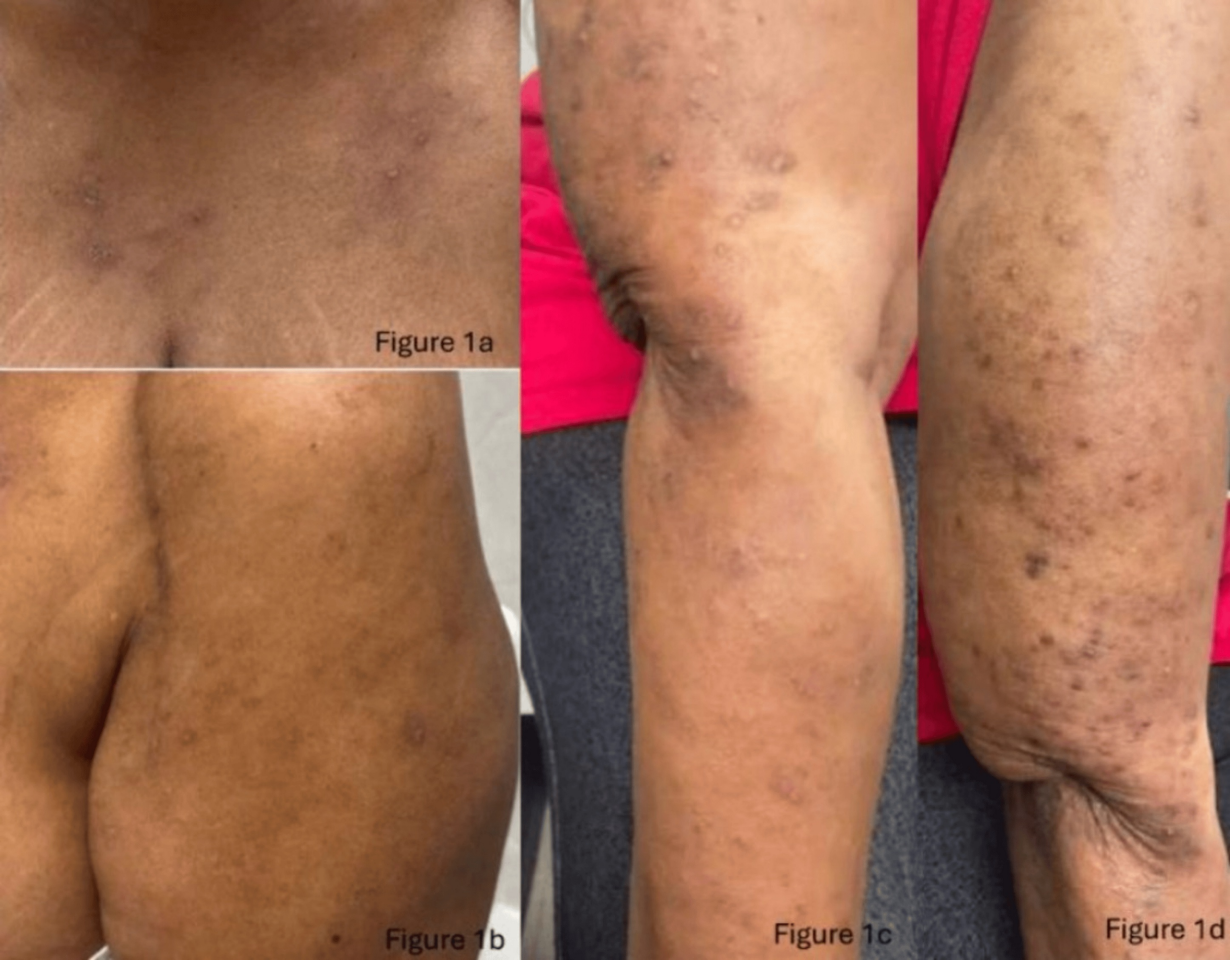
Physical exam Initial presentation (a, b). Clinical presentation post-dupilumab therapy (c, d). Hyperpigmented, hyperkeratotic papules with overlying excoriations, central areas of erosion, and admixed erythematous papules to the right upper extremity.

A 69-year-old African American female with a history of hepatitis C virus, previously treated with sofosbuvir/velpatasvir, along with premature ventricular contractions, supraventricular tachycardia, hypertension, and obesity, presented with a papular, pruritic skin eruption, initially thought to be prurigo nodularis. At this time, the patient was up-to-date on cancer screenings and denied any new systemic symptoms including fever, weight loss, or arthralgias. She presented with hyperpigmented papules with excoriated tops without evidence of blisters or bullae on the bilateral upper extremities, back, and neck secondary to reported pruritus for one to two months (Figure 1a, 1b). Over the course of several months, she underwent treatment with oral methylprednisolone, triamcinolone, and clobetasol but experienced no relief of symptoms. Given her clinical history, morphologically consistent pruritic lesions, ongoing development of new nodules, and lack of response to steroids, the initiation of dupilumab was considered appropriate for treating her prurigo nodularis, given her high lesion count.

**Figure 2:**
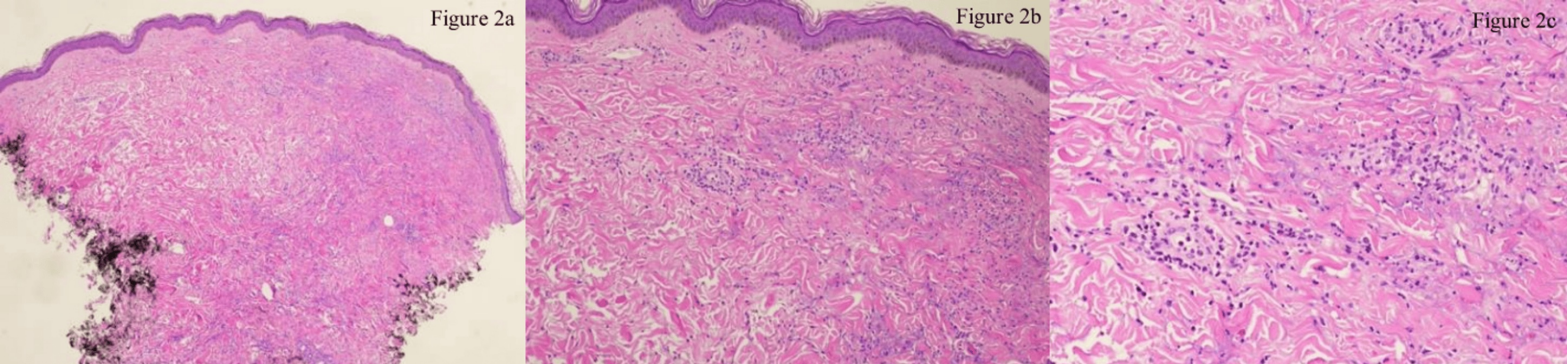
Histopathological examination of the right upper arm. Histopathology demonstrates orthokeratosis, regular acanthosis (a), and perivascular and interstitial neutrophils with numerous eosinophils, many extending into the edematous interstitium (b, c). Perivascular fibrin and leukocytoclasia present. The infiltrate extends into subcutis and is present around adnexa. This pattern is consistent with that of LCV (hematoxylin-eosin stain). LCV: leukocytoclastic vasculitis.

She was then started on dupilumab therapy, receiving a 600 mg loading dose at the initiation of treatment followed by one 300 mg dose every two weeks. Seven months later, she reported minimal improvement in her symptoms. Physical exam showed hyperpigmented, hyperkeratotic papules with central areas of erosion to the upper back and bilateral upper extremities, in addition to newly developed admixed erythematous papules to the right upper extremity which were not present previously (Figure 1c, 1d). Due to lack of improvement and the development of new, morphologically distinct erythematous papules, a punch biopsy of the right upper arm was performed. Histopathology revealed perivascular and interstitial neutrophils with numerous eosinophils extending into the interstitium, consistent with LCV with eosinophilia (Figure 2). Subsequently, dupilumab was discontinued and topical tacrolimus was initiated. One month later, she reported no new red papules aside from one small erythematous papule on her right upper extremity. At this time, another punch biopsy was performed revealing acanthosis and hyperkeratosis with thick subpapillary plate, consistent with lichen simplex chronicus with an overlying scar and suggestive of a persistent chronic inflammatory state following resolution of the initial vasculitis. 

## Discussion

We report a case of a patient with prurigo nodularis who developed LCV with eosinophilia while undergoing dupilumab therapy. LCV is a form of cutaneous small-vessel vasculitis characterized by neutrophilic infiltrate, leukocytoclasia, fibrinoid necrosis, and distinct disintegration of leukocytes, resulting in the destruction of the involved vasculature. Particularly in the context of drug-induced LCV, as these lesions progress, a mixed inflammatory infiltrate with the presence of eosinophils may result [5].

As discussed above, the initial punch biopsy revealed perivascular and interstitial neutrophils with numerous eosinophils and leukocytoclasia, consistent with LCV and suggestive of a drug-induced etiology due to the prominent eosinophilic infiltration. Additionally, given the absence of extracutaneous manifestations, further laboratory studies beyond the skin biopsy were not indicated due to low suspicion for a systemic involvement.

In this patient undergoing dupilumab therapy, although the exact pathogenesis of LCV remains indiscernible, given the histological morphology, single clinical presentation, lack of recurrence, and resolution following discontinuation of therapy, suspicion for a drug-induced pathology is high [5]. Clinical trials have revealed a transient eosinophilia associated with the introduction of dupilumab in patients. A majority of the presentations are self-resolving and clinically insignificant; however, nearly a quarter of cases with increased blood eosinophil counts have been noted to progress to significant eosinophilic diseases, including eosinophilic granulomatosis with polyangiitis (EGPA), eosinophilic pneumonia, eosinophilic vasculitis, and sudden worsening of asthma symptoms.

Although the exact mechanism remains uncertain, it is hypothesized that a multifactorial effect - driven by the initiation of a corticosteroid taper at therapy induction and the inhibition of IL-4 and IL-13 signaling pathways - leads to reduced eosinophil migration and activation. This, in turn, results in eosinophil accumulation in the blood or tissues, potentially contributing to diseases such as eosinophilic pneumonia or EGPA [4]. Additionally, a history of systemic eosinophilia, asthma, and allergic rhinitis was common amongst these patients, emphasizing the importance of accounting for additional co-occurring atopic conditions prior to the initiation of dupilumab therapy [4,6]. Given that these presentations are driven by a hyper-eosinophilic state, symptoms such as worsening asthma, sinusitis, or systemic symptoms - including fever, weight loss, arthralgias, rash, or ocular symptoms like dryness or pruritus - following dupilumab initiation should raise concern for therapy-induced eosinophilia [3,7].

Furthermore, a recent study analyzing data from the U.S. Food and Drug Administration (FDA) Adverse Event Reporting System (FAERS) database found that adverse events such as pruritus, injection site reactions, rash, and off-label use were associated with dupilumab therapy. Although most events were classified as non-serious and showed no evidence of end-organ damage, significantly elevated blood eosinophil levels were observed. These manifested clinically as allergic conjunctivitis, severe eosinophilia, and chronic eosinophilic pneumonia, ultimately leading to discontinuation of therapy [7].

## Conclusions

This patient’s findings support the need to recognize a potential association between dupilumab and the development of eosinophilic conditions such as eosinophilic granulomatosis with polyangiitis and eosinophilic LCV in the context of dupilumab therapy. The temporal relationship between the initiation of dupilumab treatment and the onset of eosinophilic LCV suggests a potential association that should be further investigated and should encourage dermatologists to remain cognizant of the development of LCV in the setting of dupilumab therapy, especially in patients with atopic conditions.
